# Effects of *Terminalia fagifolia* mart. extract combined with photobiomodulation on diabetic foot ulcers: protocol for a randomized single-blind clinical trial

**DOI:** 10.1007/s10103-026-04963-9

**Published:** 2026-07-25

**Authors:** Ana Carolina Silva Barros, Rebeca Barbosa da Rocha, Mariana Bezerra Miranda, Nathanael dos Santos Alves, Durcilene Alves da Silva, Alyne Rodrigues de Araújo Nobre, Vinicius Saura Cardoso

**Affiliations:** 1 Biomedical Master Science Program, Universidade Federal do Delta do Parnaíba, Parnaíba, Brazil; 2 Doctoral Program in Biotechnology, Universidade Federal do Delta do Parnaíba, Parnaíba, Brazil; 3 Biomedical Doctoral Science Program, Universidade Federal do Delta do Parnaíba, Parnaíba, Brazil; 4 Research Center on Biodiversity and Biotechnology, BIOTEC, Universidade Federal do Delta do Parnaíba, Parnaíba, Brazil; 5 Integrated Center of Medical Specialties, Parnaíba, Brazil

**Keywords:** Diabetic foot ulcer, Chronic wounds, Low-level laser therapy, Phytotherapy, Ulcer healing

## Abstract

Diabetic wounds are characterized by persistent inflammation, prolonged healing time, and an increased risk of amputation. The use of natural compounds combined with established therapies can increase the effectiveness of treatments for these lesions. In this context, natural products have stood out as therapeutic adjuvants due to their anti-inflammatory, antioxidant, antimicrobial, and regenerative properties. *Terminalia fagifolia* Mart., a medicinal species native to the Brazilian Cerrado, has a rich phytochemical composition; however, its clinical potential in wound healing has not yet been investigated. This is the first clinical trial to investigate the potential synergistic effects of *Terminalia fagifolia* Mart. extract, alone or in combination with photobiomodulation, on the healing of non-infected diabetic foot ulcers. A total of 102 participants with type 2 diabetes and lower limb ulcers will be randomly allocated into three groups: G1 (Terminalia fagifolia Mart. + wound occlusion), G2 (GaAs 904, 10 J/cm² + Terminalia fagifolia Mart. + wound occlusion), and a control group using Endophoton KLD (GaAs 904) at a dose of 10 J/cm² + conventional dressing. One hundred and two volunteers will be recruited and treated on two non-consecutive days per week for 10 weeks, totaling 20 sessions. The primary outcome variable will be the rate of ulcer size reduction over the 10-week period. This study is expected to provide preliminary evidence regarding the safety, feasibility, and potential efficacy of the synergistic use of T. fagifolia Mart. extract combined with photobiomodulation in the treatment of diabetic foot ulcers.

**Clinical trial registration **

This study was registered at Brazilian Clinical Trials Registry (ReBEC) on December 27, 2023 (RBR-25ng5pt || https://ensaiosclinicos.gov.br/rg/RBR-25ng5pt).

## Introducion

Diabetes mellitus (DM) is one of the leading causes of lower-limb complications. Approximately one-quarter of ulcers fail to heal, thereby increasing the risk of amputation [1]. Even when healing is achieved, recurrence rates remain high, reaching 60% at 3 years and 65% at 5 years [2]. Following amputation, the mortality rate ranges from 50% to 68% within 5 years, exceeding that associated with some types of cancer [3]. This scenario, coupled with the increased risk of amputations and recurrent hospitalizations, imposes a substantial financial burden on healthcare systems [4].

Chronic hyperglycemia prolongs the inflammatory phase and consequently impairs wound healing in patients with DM [5]. This condition hinders the proper progression to subsequent healing phases, particularly the proliferative phase, which is characterized by fibroblast migration and proliferation as well as extracellular matrix deposition [6]. In this context, natural extracts have been explored as therapeutic adjuvants, as their bioactive compounds may exert anti-inflammatory, antibacterial, antioxidant, and regenerative effects [7].

Species of the genus *Terminalia*, such as *Terminalia bellerica* and *Terminalia chebula*, demonstrate relevant effects on tissue repair, including modulation of the inflammatory response, stimulation of collagen synthesis, acceleration of epithelialization, and antimicrobial activity [8, 9]. The Brazilian species *Terminalia fagifolia* Mart., found in the Brazilian Cerrado, is popularly known as “capitão,” “capitão-do-cerrado,” “capitão-do-campo,” and “mirindiba,” and possesses broad biological potential [10–11]. Like other species of the *Terminalia* genus, *T. fagifolia* exhibits antifungal, anti-inflammatory, hypoglycemic, antibacterial, antioxidant, anticancer, hepatoprotective, and cardioprotective properties. These effects have been attributed to its diverse chemical composition, characterized by a high concentration of secondary metabolites, which confer broad therapeutic potential to the extract [12–14].

Another treatment option is photobiomodulation (PBM), a safe, effective, and low-cost therapeutic approach [15]. The primary mechanism of action of PBM is associated with mitochondrial stimulation, with cytochrome c oxidase (COX) serving as its main chromophore and playing a key role in mitochondrial respiration [16]. Light absorption by COX increases adenosine triphosphate (ATP) production, stimulates the controlled formation of reactive oxygen species (ROS), and enhances mitochondrial respiratory efficiency [17, 18]. These effects can activate several signaling pathways, such as JAK/STAT, which play essential roles in modulating inflammation and tissue repair [19].

A study conducted with 60 rats evaluated a hydrogel dressing composed of cashew gum polysaccharide (POLI) and chitosan (CHI), combined with the application of PBM at 660 nm. Animals treated with the combined intervention exhibited greater wound contraction, increased collagen deposition, reduced focal necrosis, and earlier epithelialization [20]. Gonçalves et al. [21] demonstrated that the association of PBM with *B. oleracea* resulted in a significantly greater reduction in wound area compared to the control group, indicating a possible additive or synergistic effect between the interventions.

Considering the evidence available in the literature, the combination of natural compounds with established therapies may enhance the effectiveness of diabetic foot ulcer treatment. In this context, the combination of photobiomodulation, innovative dressings, and natural compounds represents a promising strategy for the development of more effective therapies in clinical practice. To date, no study has investigated the effects of combining *T. fagifolia* Mart. extract with PBM. To our knowledge, this is the first randomized, single-blind clinical trial designed to investigate the potential synergistic effects of *T. fagifolia* Mart. extract combined with PBM in patients with DFU.

Therefore, this clinical trial will evaluate the topical application of T. *fagifolia* Mart. extract and PBM individually, as well as their combination, to investigate potential synergistic effects on the healing of DFU. Thus, the hypothesis of this study is that the synergistic effect of T. *fagifolia* Mart. extract and PBM may accelerate the tissue repair process when compared to the isolated use of each therapy.

## Methods

### Study design and recruitment

This is a randomized, single-blind experimental study with blinding applied to the evaluator. In this study, we propose a comparison between a new dressing and PBM in non-infected diabetic ulcers over a period of 10 weeks (20 interventions). *Terminalia fagifolia* Mart. sample was collected in Timon City, Maranhão State of Brazil, during a rainy summer to obtain the plant extract, that will be used at a concentration of 1 mg/mL, defined based on prior experimental optimization conducted by the research group, considering physicochemical stability and preliminary biological activity. The species was identified and the voucher deposited in the Delta do Parnaíba Herbarium (HDelta), at the Parnaíba Delta Federal University (n° 6979). Furthermore, the study was registered in the National System for the Management of Genetic Heritage and Associated Traditional Knowledge (SisGen) under number A7851F5. Detailed formulation parameters are currently under patent protection, and a comprehensive description of the standardization process will be reported in a separate study under peer review. Detailed formulation parameters are currently under patent protection, and a comprehensive description of the standardization process will be reported in a separate study under peer review. In the control group, GaAs 904 nm, 70 mW, and an energy density of 10 J/cm² (ENDOPHOTON KLD) will be used in pulsed mode, as described in a previous study [22]. This study was approved by the local Research Ethics Committee (Protocol 6.499.992) and the Brazilian Registry of Clinical Trials (ReBEC) (RBR-25ng5pt) and will be conducted in accordance with the Declaration of Helsinki and the SPIRIT [23] (Standard Protocol Items: Recommendations for Interventional Trials) and CONSORT [24] (Consolidated Standards of Reporting Trials) guidelines.

### Sample size

The sample size was calculated using G*Power 3.1.9.4 software, based on the primary outcome of wound area reduction [25]. A repeated-measures ANOVA (F-test) was used to account for the interaction between within-subject factors (three time points: baseline, 5 weeks, and 10 weeks) and between-subject factors (three groups: CG, G1, and G2). The analysis considered a statistical power of 0.90, an alpha level of 0.05, and an effect size of 0.31. Based on these parameters, a total sample size of 93 participants was estimated. Assuming a 10% loss to follow-up, 102 volunteers will be recruited (34 volunteers per group).

### Eligibility criteria

One hundred and two volunteers will be recruited through the Integrated Center for Medical Specialties (CIEM) – Polyclinic, after a detailed clinical examination of the feet. The inclusion criteria are as follows: volunteers of both sexes, aged 18 years or older, with a medical diagnosis of type 2 diabetes and diabetic ulcers located on the lower limbs. Volunteers presenting signs of infection, osteomyelitis, ischemia, inability to attend the scheduled number of sessions, use of advanced ointments or dressings, and/or any contraindication to the proposed therapeutic methods will be excluded. Written informed consent will be obtained from all eligible participants, according to the inclusion and exclusion criteria, before the start of study recruitment.

### Randomization, allocation, and blinding

**Fig. 1:**
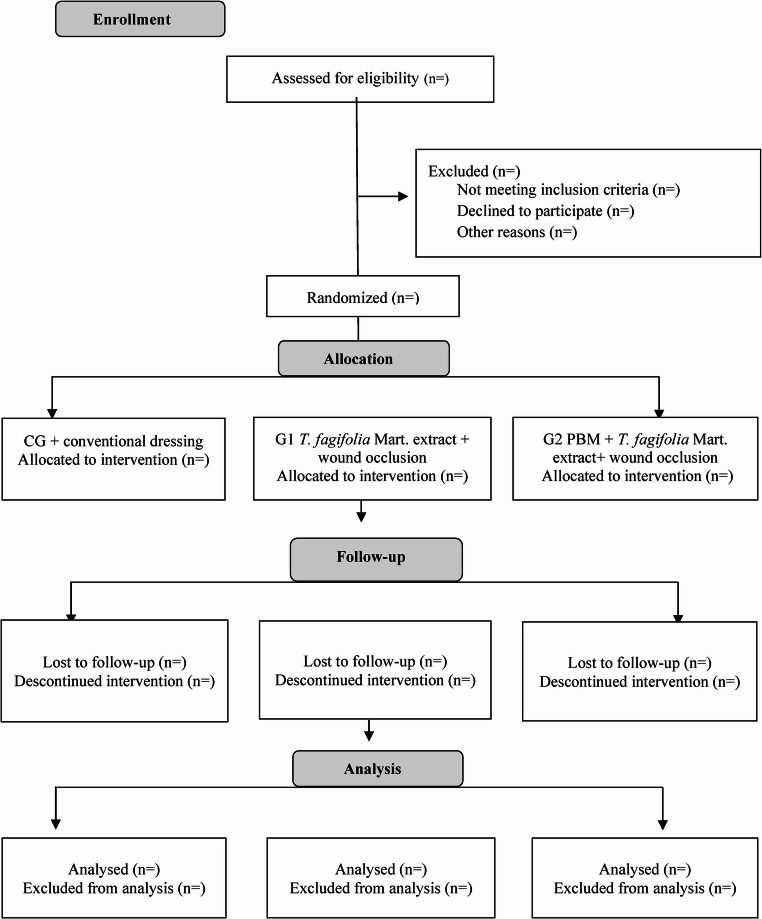
Flowchart with participant flow based on the CONSORT model. G1: *Terminalia fagifolia* Mart + wound occlusion; G2: GaAs 904, 10 J/cm^2^ + *Terminalia fagifolia* Mart.; CG: 904 nm PBM (energy density 10 J/cm^2^) + conventional curative

Participants will be assigned to one of three groups through an online randomization program [26]: G1 (*Terminalia fagifolia* Mart + wound occlusion), G2 (GaAs 904, 10 J/cm^2^ + *Terminalia fagifolia* Mart. + wound occlusion) and the control group (CG) will use Endophoton KLD (GaAs 904) with a dose of 10 J/cm^2^ + conventional dressing (Fig. 1). An independent researcher, not involved in participant care or outcome assessment, will be responsible for conducting the randomization process. Records and treatments will be placed in opaque, sealed envelopes. The study will adopt a single-blind design, in which the assessor will remain blind to the intervention received by each group until the conclusion of the clinical trial.

### Initial assessment

After meeting the eligibility criteria and signing the informed consent form, participants will have their lower limbs assessed by an external evaluator. A form developed by the researcher will be used to collect data such as personal information, clinical history, capillary blood glucose, body mass index (BMI), time since DM diagnosis, and ulcer duration. In addition, the exact affected area will be identified and recorded during the assessment.

## Product manufactured

The *T. fagifolia* Mart. extract will be combined with calcium alginate and cashew gum. After weighing, the sample will be dissolved in distilled water and subjected to solubilization. In the next step, the mixture will be measured, transferred to a container, and the solvent will be evaporated in an oven, with an average incubation time of 24 to 36 h at 40 °C. The process will be completed in a biological safety cabinet, where the sample will be sterilized using ultraviolet light, and the dressings will be stored in surgical paper and sealed. The product development process is protected by patent.

## Intervention

### Control group

The wounds will initially be cleaned with saline solution and sterile gauze. During all sessions, both the therapist and the patient will wear protective eyewear. Treatment will be performed twice a week, totaling 20 sessions over 10 weeks. The probe will be positioned perpendicularly to the ulcer, allowing precise irradiation of the wound edges. In the internal area of the wound, the sweeping technique will be applied, in which the probe will be moved slowly over the entire surface of the ulcer in continuous, parallel movements, maintaining an approximate distance of 1 cm. The duration of each session will be automatically determined by the device based on predefined energy density parameters. At the end of each application, the ulcer will be covered with a conventional dressing, defined in this study as standard wound care consisting of sterile gauze associated with topical *Helianthus annuus* vegetable oil. Patients will be instructed to clean the wound and change the dressing daily, following standardized home aseptic care guidelines [27]. To ensure protocol standardization, all sessions will be performed by the same trained therapist, using the same equipment, application method, and parameters described in Table 1.

### Intervention groups

DFUs will undergo standardized cleaning with isotonic saline solution, using aseptic technique and sterile gauze. After cleaning, group G1 will receive topical application of *T. fagifolia* Mart. extract, followed by sterile occlusion of the lesion according to the established protocol. In group G2, *T. fagifolia* Mart. extract will be applied after irradiation with PBM Endophoton KLD GaAs (904 nm, 70 mW, dose of 10 J/cm²), followed by sterile occlusion of the wound with sterile gauze. Treatment will be conducted twice a week, totaling 20 sessions over 10 weeks. To ensure protocol standardization, all sessions will be performed by the same trained therapist. Participants will be instructed to remove the previously applied extract at the end of the day, then clean the lesion with isotonic saline solution, and apply occlusion with a conventional dressing consisting of sterile gauze and *Helianthus annuus* vegetable oil on days without treatment sessions. A detailed description of the procedures applied to each group can be found in Table 2.

**Table 1:** Photobiomodulation parameters of the Endophoton KLD device used in the protocol

Parameters	PBM 904 nm	Description
Operational mode	Continuous	Device Settings
Radiant power (mW)	70 mW	Device Settings
Full power (W)	0,07 W	Device Settings
Contact area (cm^2^)	0,001 cm^2^	Device Settings
Power density (W/cm^2^)	1,00 W/cm^2^	Device Settings
Pulse duration	100 × 10^− 9^s	Device Settings
Time (s)	2s	Device Settings
Energy density (J/cm^2^)	10 J/cm^2^	Device Settings
Application technique	Scan and point	No skin contact
Irradiated area	-	Dependent on the size of the ulcer area
Number and frequency of sessions	20 sessions twice a week	-

**Table 2:** Description of therapeutic procedures in intervention groups

Procedure	Group G1 T. fagifolia extract	Group G2 PBM + T. fagifolia extract
Initial ulcer cleansing	Isotonic saline solution + aseptic technique + sterile gauze	Isotonic saline solution + aseptic technique + sterile gauze
Therapeutic application	Topical application of *T. fagifolia* Mart extract	GaAs 904 nm, 70 mW, 10 J/cm², followed by topical application of *T. fagifolia* Mart extract.
Irradiation technique	—	Probe perpendicular to the edges; scanning inside the ulcer; distance ≈ 1 cm
Wound occlusion	Sterile dressing	Sterile dressing
Frequency of sessions	2x/week	2x/week
Total sessions	20 sessions (10 weeks)	20 sessions (10 weeks)
Home care	Remove the extract at the end of the day, clean the lesion with saline solution, and cover it with a *Helianthus annuus* oil dressing.	Remove the extract at the end of the day, clean the lesion with saline solution, and cover it with a *Helianthus annuus* oil dressing.

### Primary outcome

#### Ulcer reduction rate

**Fig. 2:**
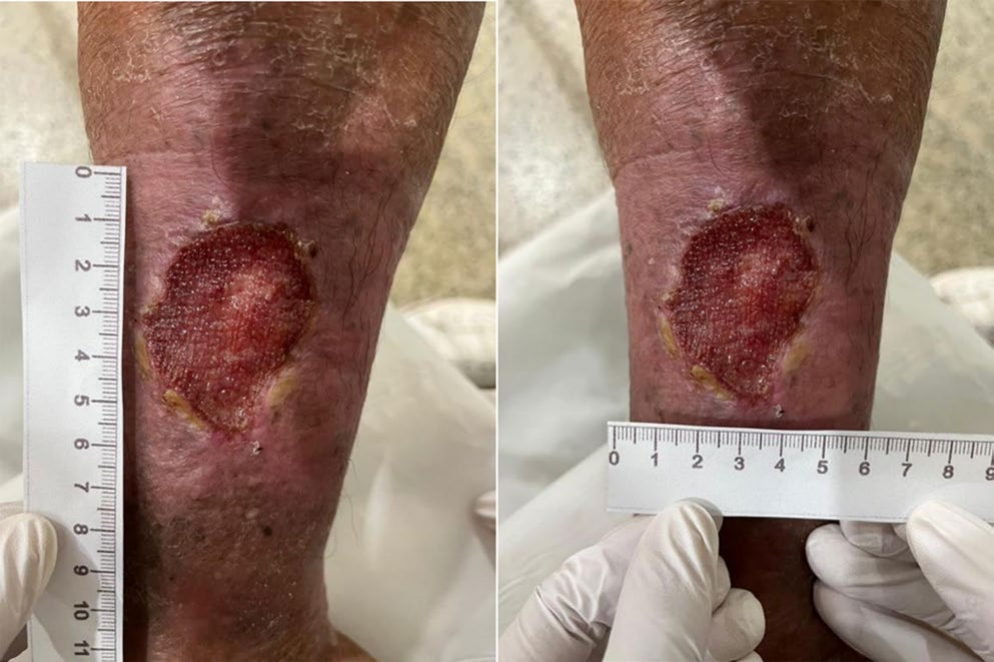
Measuring the ulcer area with a disposable paper ruler

The primary variable will be the rate of ulcer size reduction over 10 weeks. After the initial assessment, the DFUs will be identified, and the area will be photographed alongside a paper ruler, which will serve as a calibrator for the ImageJ software (Fig. 2) [28, 29]. DFUs will be assessed at three time points: pre-intervention (baseline), after 5 weeks (10 sessions), and after 10 weeks (20 sessions). The calculation of the reduction rate will follow the same parameters used by Ahmed and Irfan [30], represented by the formula: $\:\frac{Af-Ai}{Ai}\:x\:100$. Here, Ai and Af denote the initial and final ulcer areas, respectively. Ulcer area measurements will be performed by a blinded independent evaluator, who will be unaware of group allocation. The primary outcome will be compared between groups and across time points using repeated-measures analysis.

### Secondary outcomes

#### Clinical response analysis

A clinical responder analysis will be performed considering a 50% wound healing threshold, based on previously published evidence from our group and analyzed according to Farrar et al. [22, 31]. The cumulative proportion of clinical responders will be estimated across groups. For graded outcomes, the mean difference between response curves will be used to calculate the absolute risk reduction (ARR), from which the number needed to treat (NNT = 1/ARR) will be derived, providing a clinically meaningful interpretation of treatment efficacy. These outcomes will be compared between groups.

### University of Texas rankings

To identify changes in ulcer depth, ulcer images will be obtained before the intervention (baseline), after 5 weeks (10 sessions), and after 10 weeks (20 sessions), and classified using the University of Texas Wound Classification System [32]. Volunteers will be grouped into A1 and C1 (superficial ulcers), A2 and C2 (ulcers involving the tendon or joint capsule), and A3 (ulcers involving the bone). This classification will be used for longitudinal assessment of ulcer depth and compared between groups over time.

### Glycemic level assessment

Participants random blood glucose which will be measured levels will be monitored once per week throughout the 20 sessions using the Accu-Chek^®^ Active glucometer. Patients will be instructed to keep their arms extended alongside their bodies, in a vertical position, for approximately one minute. A puncture will then be performed using sterile lancets, and the blood sample will be applied to the device’s specific reagent strip. Glycemic levels will be analyzed to assess potential associations with wound healing and compared between groups over time.

### Data monitoring

This is the first clinical trial to evaluate the topical application of *T. fagifolia* Mart. extract in ulcers, providing initial data on its safety in humans. All procedures related to ulcer assessment, extract application, and adverse event reporting will be prospectively recorded using standardized forms. Safety monitoring will include active and systematic surveillance of local reactions at the lesion site (erythema, pain, pruritus, increased exudate, and signs of infection), as well as systemic adverse events potentially associated with the intervention. All events will be documented, classified according to severity and causality, and managed in accordance with predefined clinical protocols. Serious or unexpected adverse events will be promptly reported to the Research Ethics Committee, in compliance with current national regulations.

Participants will be monitored at each treatment session for signs of secondary infection throughout the study. At each session, lesions will be systematically assessed by a trained evaluator for clinical indicators of infection, including purulent exudate, malodor, dark discoloration, tissue necrosis, perilesional cellulitis, and systemic signs such as fever. The diagnosis of infection will follow the criteria established by the Infectious Diseases Society of America (IDSA) [33], requiring the presence of at least two local signs of inflammation or infection. In cases where infection is suspected or confirmed, the participant will be promptly referred to an independent physician, not involved in the intervention, for appropriate clinical management. The intervention protocol may be adjusted or discontinued if clinically indicated to ensure patient safety (Table 3).

**Table 3:** Recruitment, interventions, and assessment schedule according to the recommendations for interventional trials (SPIRIT)

STUDY PERIOD							
	Enrollment		Post-allocation (weeks)				Post-intervention
TIMEPOINT	Recruitment	Allocation	Basiline	5 W	10 W	Once a week	t_x_
ENROLLMENT:							
* Recruitment*	X						
* Selection Criteria*	X						
* Allocation*		X					
INTERVENTIONS							
* CG*			X	X	X		
* G1*			X	X	X		
* G2*			X	X	X		
ASSESSMENTS:							
* Evaluation form*			X				
* Ulcer reduction rate*			X	X	X		
* Clinical Response Analysis*			X	X	X		
* Glycemic level assessment*			X			X	
* University of Texas Rankings*			X	X	X		

### Statistical analysis

All statistical analyses will be conducted according to the intention-to-treat principle. Data will be tabulated in spreadsheets (Excel package) and analyzed using SPSS version 29 (Statistical Package for the Social Sciences). Sociodemographic data will be presented as descriptive statistics, absolute values and percentages, or mean and standard deviation (median and interquartile range). Normality will be assessed using the Kolmogorov–Smirnov test. The chi-square test will be applied for categorical data. To analyze differences between groups, a one-way ANOVA or the Kruskal–Wallis test will be performed. Intragroup analysis will be conducted using one-way repeated-measures ANOVA or the Friedman test. Statistical significance will be set at *p* < 0.05, and results will be presented in tables. The cumulative proportion of responders will be calculated considering a 50% healing rate, based on the article previously published by the group, and analyzed as described by Farrar et al. [22, 31]. In studies with graded outcomes, the mean difference between the curves of two groups represents the absolute risk reduction (ARR), from which the number needed to treat (NNT = 1/ARR) can be derived.

## Discussion

This is the first randomized, single-blind clinical trial to investigate the effects of combining *T. fagifolia* Mart. extract with PBM on the healing of DFUs. Chronic hyperglycemia impairs leukocyte function, compromises microcirculation, and alters the inflammatory response, resulting in prolonged and ineffective healing phases [34]. In clinical practice, the healing of DFUs poses significant challenges, with a substantial risk of infections and amputations [35]. These complications impair gait, reduce quality of life, and increase costs associated with prolonged hospitalizations, representing one of the major public health challenges [36].

From a health economics perspective, although this study does not include a formal cost-effectiveness analysis, the proposed interventions may have substantial implications for healthcare systems, particularly in resource-limited settings such as Brazil [37]. The management of diabetic foot ulcers imposes a significant economic burden, driven by prolonged treatment duration, high recurrence rates, frequent hospitalizations, and the risk of amputation [1, 36]. In this context, PBM has emerged as a low-cost, non-invasive, and operationally feasible therapy, while the use of *T. fagifolia* Mart. extract represents a potentially scalable and locally accessible therapeutic strategy [15, 41]. The combination of these approaches may enhance clinical efficiency by accelerating wound healing and reducing complication rates, thereby contributing to a reduction in overall healthcare expenditures.

The topical application of plant extracts has been widely explored for wound healing due to their bioactive compounds, which can provide anti-inflammatory, antioxidant, antimicrobial, and tissue-regenerating effects [38]. In preclinical models, several extracts from medicinal plants have been shown to accelerate wound closure, increase collagen deposition, stimulate angiogenesis, promote fibroblast proliferation, enhance epithelialization, and reduce the microbial load in lesions [39, 40].

In this context, species of the genus *Terminalia* have been extensively investigated due to their medicinal properties [41]. Among them, *T. arjuna*, *T. bellerica*, *T. catappa*, and *T. chebula* have demonstrated the potential to accelerate wound healing in animal models, promoting higher contraction rates, reduced epithelialization time, and decreased lesion area [42–45]. Furthermore, Rodrigues de Araújo et al. [14, 46] reported that *T. fagifolia* Mart. extracts exhibit antibacterial, antibiofilm, and cytotoxic activities, highlighting their therapeutic potential in controlling infections, particularly those associated with ulcers and bacterial biofilms.

Photobiomodulation has emerged as another therapeutic alternative, demonstrating consistent results, particularly in accelerating healing and reducing associated complications [25]. Saura et al. [22], in a clinical trial, showed that PBM using a 904 nm wavelength and a dose of 10 J/cm² promoted a significant reduction in the area of diabetic ulcers after 10 weeks when compared with conventional treatment. Another clinical trial [47] demonstrated that, after four weeks of application of GaAs 904 nm (2 J/cm²), there was a significant reduction in VEGF compared with placebo, as well as a negative correlation between VEGF and %DWSA, indicating an improvement in the ischemic condition of the lesions.

Other authors have demonstrated that the use of PBM in combination with plant extracts can enhance individual outcomes and promote improved tissue repair [48] The combination of PBM with homeopathic preparations of plant extracts, such as *Calendula officinalis*, *Hypericum perforatum*, and *Echinacea purpurea*, has been shown to improve wound healing in experimental models [49]. The interaction between plant extracts and PBM may occur at different levels. These extracts contain phenolic and flavonoid compounds with anti-inflammatory and antioxidant effects, which complement the effects of PBM in reducing inflammation and promoting tissue repair [50].

In this context, the combination of T. *fagifolia* extract with photobiomodulation (PBM) may present promising synergistic effects in tissue repair. The bioactive compounds present in the plant extract can exert antioxidant, antimicrobial, and anti-inflammatory activity, contributing to the reduction of oxidative stress, microbial load, and persistent intensity, critical factors in delaying healing in diabetic foot ulcers [14, 46]. These actions favor the restoration of a more balanced microenvironment conducive to tissue repair. In parallel, PBM stimulates fundamental cellular processes for healing, including mitochondrial activation with increased ATP production, fibroblast strategy, type I collagen synthesis, angiogenesis, and epithelial cell migration, as well as the modulation of inflammatory mediators and extracellular matrix metabolism [51, 52].

Thus, the biological effects of the extract can act in a complementary way to PBM, reducing adverse conditions in the wound microenvironment, such as excess reactive oxygen species and microbial presence, enhancing the cellular response caused by photobiomodulation. This interaction can favor greater efficiency in tissue repair processes, promoting more organized and functional healing.

Therefore, this clinical trial will evaluate the topical application of *T. fagifolia* Mart. extract and PBM individually, as well as their combination, to investigate potential synergistic effects on the healing of diabetic foot ulcers. It also aims to assess not only the efficacy but also the safety and clinical feasibility of this therapeutic approach as an accessible and applicable resource in clinical practice.

Although wound area reduction and complete healing are the primary outcomes of this study, future investigations should incorporate additional clinical and laboratory parameters, including collagen deposition, granulation tissue formation, tissue elasticity, pain assessment, nutritional and inflammatory biomarkers, as well as analyses of pro-inflammatory cytokines and ultrastructural assessments. These measures may provide a more comprehensive understanding of tissue remodeling, inflammation resolution, infectious status, metabolic control, nerve recovery, and the long-term durability of wound repair.

### Study limitations

This study presents some limitations that should be acknowledged. The single-blind (evaluator-blinded) design may introduce a potential risk of bias. The implementation of a double-blind design was limited by the characteristics of the interventions, as both topical application of *T. fagifolia* Mart. extract and photobiomodulation involve visible and distinct procedures, making participant and therapist blinding difficult. However, important methodological strategies will be adopted to minimize potential bias, including blinded outcome assessment, random allocation, and standardized intervention protocols conducted by trained professionals. These measures strengthen the internal validity and reliability of the results. Furthermore, HbA1c was not included in this protocol due to resource limitations. Future studies should incorporate HbA1c assessment to provide a more comprehensive evaluation of long-term glycemic control and its relationship to wound healing outcomes, and should consider double-blind designs, when feasible, to strengthen the internal validity of the findings.

## Data Availability

No datasets were generated or analysed during the current study.

## Declarations

### Permission to reproduce material from other sources

This manuscript does not contain any previously published material from the sources.
